# Inhibition of endoplasmic-reticulum-stress-mediated autophagy enhances the effectiveness of chemotherapeutics on pancreatic cancer

**DOI:** 10.1186/s12967-018-1562-z

**Published:** 2018-07-09

**Authors:** Prakash C. Thakur, Jennifer L. Miller-Ocuin, Khanh Nguyen, Rina Matsuda, Aatur D. Singhi, Herbert J. Zeh, Nathan Bahary

**Affiliations:** 1 Division of Hematology/Oncology, Department of Medicine, University of Pittsburgh Cancer Institute, UPMC Hillman Cancer Center, Pittsburgh, PA USA; 2 Department of Surgery, University of Pittsburgh School of Medicine, Pittsburgh, PA USA; 3 Department of Pathology, University of Pittsburgh Medical Center, Pittsburgh, PA USA; 4 Present Address: Smilow Cancer Hospital, Yale School of Medicine, New Haven, CT USA; 5 Present Address: University of Pennsylvania, Perelman School of Medicine, Philadelphia, PA USA

**Keywords:** PDAC, ER stress, UPR, Autophagy, Pancreatic cancer

## Abstract

**Background:**

Endoplasmic reticulum (ER) stress and its consequent unfolded protein response (UPR) are believed to be associated with progression, survival and chemoresistance of a variety of tumor cells through multiple cellular processes, including autophagy. Therefore, the ER stress-autophagy pathway presents a potential molecular target for therapeutic intervention. The objective of this study was to evaluate the therapeutic efficacy of ER stress and autophagy modulators in the context of pancreatic ductal adenocarcinoma (PDAC).

**Methods:**

We first targeted IRE1α, an important regulator of the UPR, through STF-083010 treatment in PDAC cell lines in vitro. Chloroquine was then used to target autophagy and an optimal combination treatment was developed using chloroquine, sunitinib and gemcitabine. Apoptosis was analyzed using TUNEL assay, autophagy was estimated using lysotracker staining and electron microscopy, and UPR was analyzed using anti-GRP78 immunostaining and XBP1 splicing. Transplantation of PDAC derived KPCP1 and Panc02 cells in mouse pancreas were performed to study treatment efficacy in vivo.

**Results:**

Suppression of the IRE1α by STF-083010 alone resulted in increased lysosomes and reduced viability of PDAC cells. Chloroquine treatment alone inhibited downstream autophagy but was insufficient in reducing PDAC cell growth. However, combining STF-083010 and chloroquine had additive anti-tumor efficacy when used with gemcitabine. Sunitinib alone caused abnormal maturation of the autolysosomes with increased intracellular multivesicular bodies and increased apoptosis evident in PDAC cells. Sunitinib showed a synergistic effect with chloroquine in reducing in vitro PDAC cell viability and significantly increased the efficacy of gemcitabine in human and murine PDAC cell lines. The anti-proliferative effect of gemcitabine was significantly increased when used in combination with sunitinib and/or chloroquine in both in vitro and in vivo PDAC models. The addition of sunitinib and/or chloroquine to gemcitabine, resulted in a significantly increased survival of the animals without noticeably increased toxicity. Sunitinib, gemcitabine and chloroquine treated mice showed a significant reduction of GRP78 expression, reduced cell proliferation and increased apoptosis in pancreas, compatible with a tumor response.

**Conclusions:**

Sunitinib combined with chloroquine reduces tumor growth through suppression of autophagy and increased apoptosis. Co-administration of modulators of ER stress-mediated autophagy with chemotherapy presents a novel therapeutic approach in PDAC.

**Electronic supplementary material:**

The online version of this article (10.1186/s12967-018-1562-z) contains supplementary material, which is available to authorized users.

## Background

Pancreatic ductal adenocarcinoma (PDAC) is a leading cause of cancer related death. Often diagnosed at an advanced stage, the average 5-year survival rate is 6% or less [1]. Pancreatic cancer currently ranks as the seventh leading cause of cancer deaths globally and the third in the United States; it is projected to become the number two leading cause of cancer deaths by the year 2020, as reported by the American Cancer Society (*Cancer Facts and Figures 2017*). Unfortunately, although surgical resection is the only curative option, more than 80% of PDAC patients are diagnosed with unresectable disease with an average survival of 12–18 months [2, 3]. Historically, standard chemotherapy has consisted of gemcitabine or 5-fluorouracil, although more recent combinations including FOLFIRINOX and nab-paclitaxel with gemcitabine have been associated with incremental improvements in survival [4–6]. The lack of more successful treatments makes novel therapeutic options desirable. As one example, combining chemotherapeutics and drugs targeting endoplasmic reticulum (ER) stress and the autophagy may be a potential avenue to explore novel therapeutic combinations.

The ER performs crucial biosynthetic and signaling functions in eukaryotic cells, including vesicular trafficking, intracellular calcium homeostasis, synthesis, folding and modifications of secretory and membrane proteins [7, 8]. These processes are assisted and monitored by ER resident chaperones and calcium-binding proteins, such as GRP78 (also knowns as HSPA5 or BIP). Various pathophysiological conditions, including hypoxia, oxidative stress, and glucose deprivation can perturb ER homeostasis and cause an imbalance between ER protein-folding load and capacity, leading to accumulation of unfolded proteins in the ER, a condition known as “ER stress” (Additional file 1: Figure S1). This in turn activates an evolutionarily conserved, integrated signal transduction pathway, termed the unfolded protein response (UPR) [9]. The UPR pathway essentially re-establishes ER homeostasis, primarily by ameliorating the protein load in the ER and reducing protein translation. This occurs through a complex transcriptional program mediated by the three distinct arms of ER stress mediators: IRE1α/XBP1, PERK/EIF2α and ATF6 to increase ER folding capacity and ER-associated degradation (ERAD), as well as via adaptive mechanisms involving the stimulation of pro-survival autophagy and auto-lysosomal degradation [7, 10, 11]. GRP78 is a crucial regulator of UPR and normally binds to IRE1α under normal physiological state (Additional file 1: Figure S1). Under ER stress, it releases IRE1α, which leads to oligomerization and trans-autophosphorylation of IRE1α. The activated IRE1α cleaves the *XBP1* mRNA into its active spliced form (*XBP1s*), which becomes a transcription factor for various UPR genes that helps in autophagy or apoptosis (Additional file 1: Figure S1) [7].

The UPR is classically linked to the maintenance of cellular homeostasis in specialized secretory cells, such as pancreatic and immune cells, in which the high demand for protein synthesis and secretion constitutes a constant source of proteostasis and cellular stress. Pancreatic cells have high hormone and enzyme secretory functions and have highly developed ER. Pancreatic cancer is also extremely rich in stroma, is hypoxic and deficient in metabolites [12]. Tumor cells are confronted with chronic metabolic stress conditions that favor the activation of adaptive mechanisms, such as the UPR and autophagy [13, 14]. The role of ER stress in pancreatic cancer pathobiology and inflammation has been increasingly recognized as an important factor in tumorigenesis and chemoresistance [15]. Moreover, certain anti-cancer therapeutics may chronically activate ER stress and autophagy, and such a drug-induced ER stress leads to the pro-survival response of the cancer cells, which consequently allows tumors to develop non-responsiveness to a particular chemotherapeutic [16, 17]. Hence, the dynamics of UPR and autophagy/lysosomal pathways present potential therapeutic targets. The molecular link between the UPR and the autophagic response to ER stress, and how these stress pathways influence therapeutic outcome and chemoresistance, remain largely undefined, making this topic highly imperative for preclinical and clinical cancer research.

Recently, a variety of anti-cancer therapies have been linked to the induction of ER proteostasis in cancer cells, suggesting that strategies devised to stimulate its pro-death function or block its pro-survival function, could be envisaged to improve their tumoricidal action [18]. Previous reports as well as our current study have shown that GRP78, a critical regulator of ER stress, is enriched in the invasive ductal component as well as the surrounding stroma in PDAC tissue [19]. ER stress and the UPR pathway can be modulated by chemotherapy and other compounds. For instance, tunicamycin can block protein glycosylation and cause accumulation of unfolded proteins in the ER and hence trigger the UPR. IRE1α oligomerization, required for upregulation of the UPR, can be inhibited by a small compound, STF-083010, which subsequently blocks *XBP1* splicing activity (Additional file 1: Figure S1) [20]. STF-083010 is shown to induce tumor apoptosis and reduce growth of multiple myeloma in preclinical studies [20]. It has been hypothesized that the IRE1α auto-phosphorylation can be presumably inhibited by other kinase inhibitors. Sunitinib, a multi-tyrosine kinase inhibitor, is presumably believed to affect IRE1α autophosphorylation as well as lysosomes (Additional file 1: Figure S1), although the mechanisms are not known [21, 22]. Sunitinib is clinically approved for treating several solid tumors, including, pancreatic neuroendocrine cancer. Furthermore, sunitinib in combination with gemcitabine has been explored for advanced solid tumors in a phase-I clinical study [23, 24].

A better understanding of the molecular mechanisms that determine the outcome of UPR and autophagy activation by chemotherapeutic agents, will offer new opportunities to improve existing cancer therapies as well as unravel novel targets for pancreatic cancer treatment. We hypothesize that inhibiting the protective mechanism of the PDAC cells by modulators of UPR, autophagy and lysosomal degradation, will suppress cancer cell proliferation and induce cell death. Therefore, we sought to analyze the combinatorial effects of selected modulators of ER stress and autophagy along with gemcitabine in PDAC cells and animal models.

## Methods

### Cell lines and cell culture

The human PDAC cell lines Panc02.03, Panc3.27, Miapaca-2, and the murine PDAC cell lines, Panc02, and KPCP1 were originally procured from ATCC (Manassas, VA). Miapaca-2 was cultured in DMEM medium, and the rest others were cultured in ATCC-recommended RPMI-1640 supplemented with 10% fetal bovine serum and maintained at 5% CO_2_ at 37 °C. For long-term storage, the cells were frozen in a 5% DMSO containing the respective tissue culture medium in liquid nitrogen. Cell viability assays were carried out using Trypan-blue exclusion method using Beckman Coulter Vi-CELL™ cell viability analyzer and Image analysis [25].

### Cell-based drug assays

The following drugs were used in this study: Tunicamycin (Sigma-Aldrich) was prepared fresh in DMSO media for 5 mM stock solution. STF-083010 (Sigma-Aldrich) was prepared fresh in dark room with DMSO for 25 mM stock solution. 4-Phenylbutyric acid, sodium salt (Sigma-Aldrich) was dissolved in water at 100 mM stock solution. Chloroquine (Sigma-Aldrich) was prepared fresh in water at 50 mM stock solution. Gemcitabine and taxol solutions were freshly prepared in aliquots of 5 mM for one-time usage. Sunitinib maleate salt (Sigma-Aldrich) was dissolved in DMSO in dark room at 5 mM stock solution.

About 10,000 cells were seeded onto 12-well microtiter plates and allowed to attach overnight. Drug treatments typically started at about 50% confluence for 72 h incubation and dosing. After the drug treatment, cells were washed 2 × with fresh culture media and trypsinized (0.15% Trypsin, Invitrogen) for cell viability assays. For lysosome staining, 50 nM of lysotracker dye (LysoTracker™ Red DNN-99, Invitrogen) was added to the wells and the live cells were incubated for 45 min followed by 3 × washes with tissue culture media and imaged by fluorescent microscopy (Zeiss Axiovert) and quantified using ImageJ [26]. For TUNEL assays, cells were seeded onto sterile 8-chamber borosilicate cover glass (Tissue-Tek) and after treatment, cells were fixed with 4% PFA for 2 h, followed by the TUNEL protocol recommended by the Cell death detection kit, Fluorescein (Roche) [27]. In brief, the fixed cells were washed with PBS, permeabilized with freshly prepared 0.1% Triton X-100 and 0.1% sodium citrate for 2 min on ice, followed by washing with PBS. The cells in Tissue-Tek glass chambers were then overlaid with 100 μl TUNEL reaction mix, according to manufacturer’s instructions and incubated at 37 °C for 1 h, washed again with PBS and mounted with anti-fading reagent with DAPI (Molecular Probes) and imaged using Zeiss Axiovert with 488 nm filter. The TUNEL positive cells were quantified using ImageJ. Statistical analysis was performed by using *t*-test, and a *p*-value < 0.05 was considered significant.

### RT-PCR

Total mRNA was extracted from cell pellets using Exiquon™ cell RNA kit, and the concentration was measured using spectrophotometer. A consistent amount of template mRNA was used for RT-PCR assay using Ambion™ RT-PCR kit, following manufacturer’s protocol. The PCR products were subjected to 2% Agarose electrophoresis in TBE buffer and the gel was imaged using Bio-Rad gel documentation unit.

Primers used for *GRP78*:

Forward, 5′-CCAAGAGAGGGTTCTTGAATCTCG-3′

Reverse, 5′-ATGGGCCAGCCTGGATATACAACA-3′

Primers used for *XBP1*:

Forward, 5′-GGAGTTAAGACAGCGCTTGGGGA-3′

Reverse, 5′-TGTTCTGGAGGGGTGACAACTGGG-3′

### Animal experiments

Female C57BL/6 (B6, H-2^b^) mice, 8–10 weeks old, were purchased from Taconic (Germantown, NY). Animals were maintained in a specific-pathogen-free facility at the University of Pittsburgh Cancer Institute in accordance with the Institutional IACUC and NIH guidelines. Cell suspension of KPCP1 or Panc02 were prepared to a concentration of 1 million cells in 20 µl PBS solution. Pre-weighted mice were anesthetized, and a small incision of 1-cm was created at the left abdominal flank medial to the splenic silhouette. The pancreas was gently exposed and the cell suspension was injected under direct visualization into the pancreatic tail. The abdominal muscle and skin layer were subsequently sutured. Mice were monitored until 2 weeks for palpable tumor growth and general signs of morbidity. After 2 weeks of tumor growth, the drug treatment was initiated using 10 mice for each treatment group. At first the chloroquine (50 mg/Kg) was administered daily by intraperitoneal injection, sunitinib (25 mg/Kg) was administered daily by oral gavage, and Chemotherapy (Chemo) containing gemcitabine (25 mg/Kg) plus paclitaxel (10 mg/Kg) was intraperitoneally injected once weekly. Treatment was continued until the mice were alive for the survival analysis. For the tissue corollary study, 3 mice from each treatment group were sacrificed after 4-weeks of treatment, and pancreatic tissues were surgically removed and fixed in 4% PFA/PBS solution. All animal experiments were conducted in strict adherence with the Institutional IACUC and NIH guidelines. Statistical analysis was performed by using *t*-test, and a *p*-value < 0.05 was considered significant. The Kaplan–Meier method and log-rank test were used to evaluate the survival analysis.

### Histology and immunostaining

Human PDAC tissue sections from different tumor grades, pancreatitis tissue and normal healthy pancreatic tissue sections were obtained from the Department of Pathology, University of Pittsburgh Medical Center. PDAC Tissue microarrays with normal adjacent tissues were purchased from US Biomax (Catalog # PA241d). Mouse tissue samples were removed for corollary studies and fixed in 4% PFA in PBS, processed and embedded in paraffin. All paraffin-embedded tissues were sectioned at 5 µM sections. Sequential sections were stained with Hematoxylin and Eosin (H&E) or left unstained for IHC studies. For the quantitative analysis of apoptosis, paraffin-embedded tissue sections were assayed using TUNEL method standardized at the UPCI Tissue and Research Pathology resources. Anti-active Caspase-3 (Abcam) IHC was performed to further analyze apoptosis in murine tissues. Statistical analysis was performed by using *t*-test, and a *p*-value < 0.05 was considered significant. For cell proliferation assays, anti-Ki67 (Abcam) IHC was performed on alternate sections and imaged using Zeiss Axiovert microscope. TUNEL-positive cells, active Casp-3 positive cells and Ki67-positive cells were counted in viable regions of the ductal carcinoma in the pancreas in 5 random 20 × fields and expressed as percentage of the total cells counted in the foci. For each corollary study, representative tissue sections from 3 mice were used. Statistical analysis was performed by using *t*-test, and a *p*-value < 0.05 was considered significant. GRP78 expression was analyzed by IHC using respective anti-GRP78 antibodies (Abcam) specific to mouse or human for the respective tissue sections and the intensity of immunostaining was analyzed in both ductal carcinoma and adjacent acini and surrounding inflammatory regions. Intensity of anti-GRP78 staining was ranked as weak (scores: 1–2) or strong (scores: 3–4), and the Mann–Whitney *U*-test was used to compare GRP78 expression levels in the PDAC tissues and the non-tumor adjacent tissues (NAT) and a *p*-value < 0.05 was considered significant.

## Results

### The ER stress sensor GRP78 is elevated in pancreatic tissue of PDAC patients

**Fig. 1:**
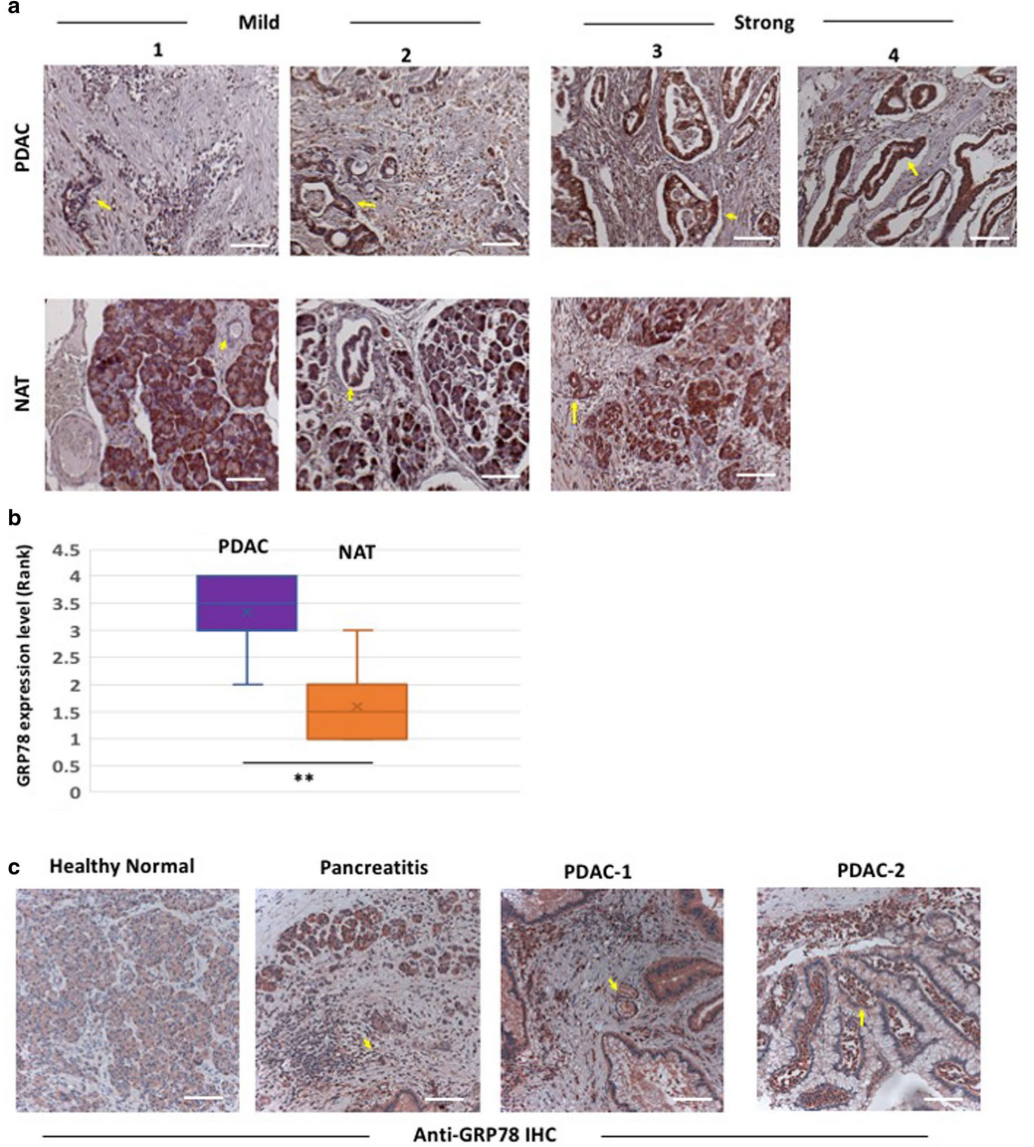
Anti-GRP78 immunostaining in PDAC tissue microarray and patient-derived pancreatic tissue biopsies. **a** Representative images of Anti-GRP78 immunohistochemistry (red chromogens) on PDAC tissue microarray showing different levels of GRP78 expression. PDAC tissues show consistently higher GRP78 expression (*arrow*) compared to non-tumor adjacent tissues (NAT). **b** Bar-charts showing increased level of GRP78 expression in PDAC tissues (*p* < 0.05, Mann–Whitney *U*-test). **c** IHC with anti-GRP78 antibody on biopsied pancreatic tissue samples from patients with a normal healthy pancreas, pancreatitis, and two separate cases of pancreatic tumors (PDAC-1 and PDAC-2). Enriched expression of GRP78 is evident in the invasive ductal adenocarcinoma cells (*arrow*) and the surrounding inflammatory stromal cells (*arrowhead*) in the tumor samples. GRP78 expression is also elevated in acini (*arrow*) and inflammatory cells (*arrowhead*) in pancreatitis, as compared to the minimal basal expression in healthy pancreas. Scale bar: 10 µm

To assess the ER stress and UPR activity in the tumor microenvironment, we analyzed 24 PDAC core tissues obtained in tissue microarray, representing six different tumor cases. We examined the expression of the proximal ER stress sensor GRP78 by anti-GRP78 immunohistochemistry (IHC) on the tumor tissues and the non-tumor adjacent tissues (NAT). These studies revealed elevated expression of GRP78 in pancreatic ductal tissues of PDAC compared to normal healthy tissues (*p* < 0.05, Fig. 1a, b). We further examined tissues exhibiting different grades of PDAC (2 cases prior to neo-adjuvant therapies), pancreatitis (1 case) and apparently healthy pancreatic tissue (2 cases) obtained from routine human tissue biopsies in our clinic. GRP78 staining was significantly enriched in the invasive PDAC cells as well as the surrounding PDAC stromal cells in both the cases of PDAC (Fig. 1c). GRP78 was also elevated in the acinar and inflammatory cells of the tissues exhibiting pancreatitis pathology (Fig. 1c).

### Pharmacological modulation of ER stress and UPR alters autophagy in pancreatic cancer cells

**Fig. 2:**
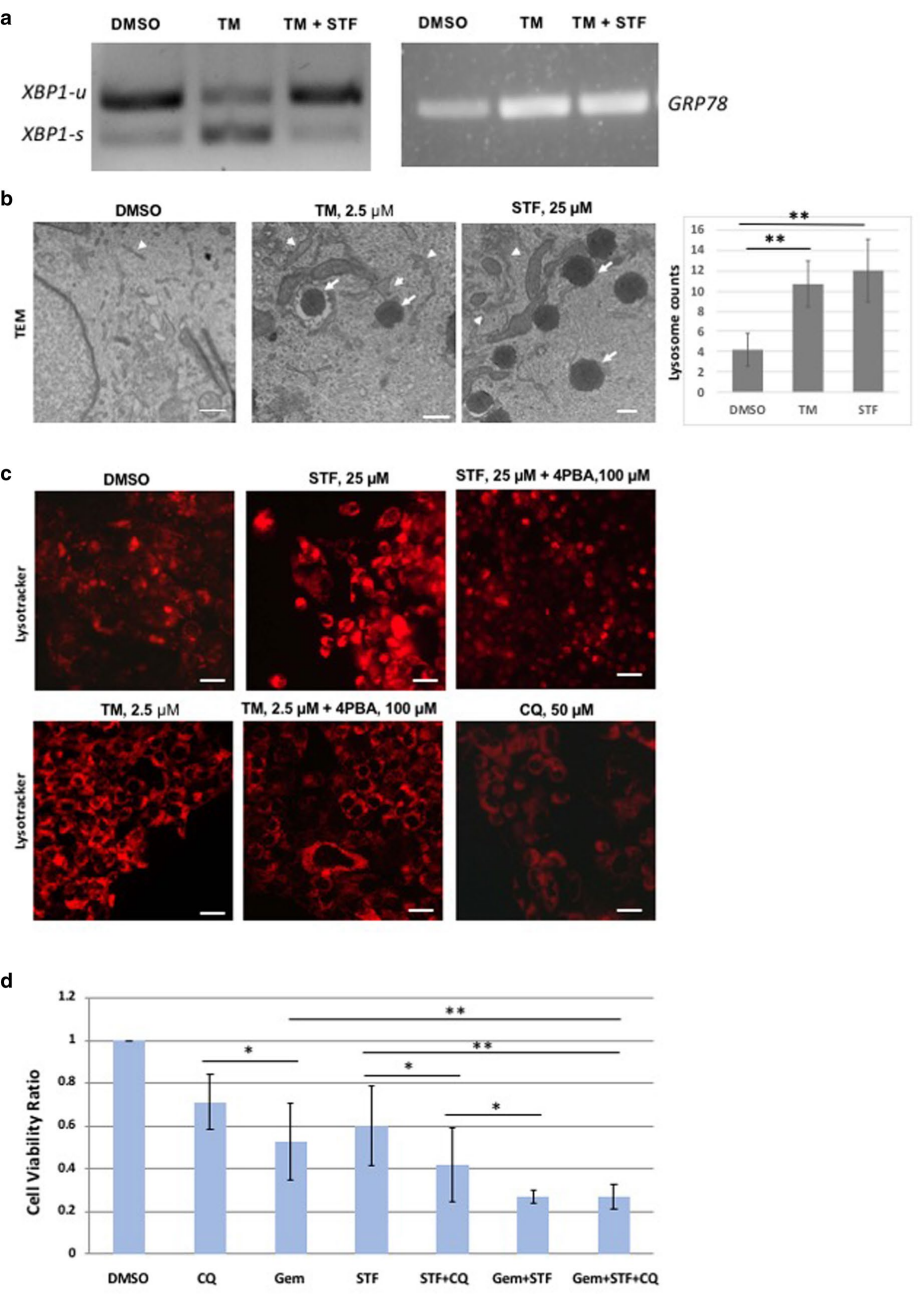
*XBP1* splicing, lysosomal activities and cell viability in human PDAC cells treated with ER stress and autophagy modulating drugs. **a** Human PDAC derived Panc02.03 cells were treated with 2.5 µM tunicamycin and/or 25 µM STF for 72 h, and RT-PCR was performed on the extracted mRNA of these cells to monitor the *XBP1* splicing and *GRP78* expression. As expected, *XBP1* splicing is increased in tunicamycin treated cells compared to DMSO treated cells, while co-treatment of STF-083010 reverses the upregulated splicing to the normal splicing levels, comparable to that of DMSO treated cells. *GRP78* is increased with tunicamycin treatment, but is not further altered by the addition of STF-083010 treatment. **b** TEM images of DMSO (left) and tunicamycin-treated (middle) and STF-083010 (right) Panc02.03 cells, showing organelle pathology of ER vesicles (arrowhead), and lysosomes (arrow). Both tunicamycin and STF-083010 treated cells exhibit enlarged ER vesicles and have increased lysosomes in the cytoplasm. Bar-charts shows significantly increased lysosome contents in STF-083010 treated cells. Scale bar, 500 nM. **c** Panc02.03 cells were treated with tunicamycin (2.5 µM), STF-083010 (25 µM) with or without 4-PBA (100 µM) for 72 h, and the lysosomes were viewed by fluorescent microscopy using lysotracker staining. Both STF-083010 and tunicamycin treatments led to increased lysotracker staining, compared to DMSO treatment groups. Co-treatment with 4PBA reduced the elevated lysotracker staining to a comparable level with the DMSO treated group. Chloroquine at 50 µM causes cell clumping and cell death and reduced lysotracker staining. Scale bar: 5 µm. **d** Bar-charts showing normalized cell viability ratio of PDAC cells treated with STF-083010 (25 µM), chloroquine (50 µM), gemcitabine (250 nM) and various combinations (n = 4). Human PDAC-derived Panc02.03 cells were treated with various concentrations of single or combination treatments for 72 h and the viability was measured by Trypan Blue exclusion assay. STF-083010 and chloroquine show additive effects with gemcitabine (*p* < 0.01). Cell viability ratio was normalized with the viability of DMSO treated cells. *p < 0.05, **p < 0.01, *CQ* chloroquine, *STF* STF-083010, *TM* tunicamycin, *Gem* gemcitabine

We used several human PDAC cell lines carrying *KRAS* mutations, namely, Panc02.03, Miapaca-2 and Panc03.27 to examine the effect of dysregulated ER stress on autophagy and cell viability. Tunicamycin inhibits protein glycosylation and causes accumulation of unfolded proteins in ER lumen, thus triggering ER stress and constitutive upregulation of UPR pathways [28]. STF-083010, on the other hand, targets the IRE1alpha arm of UPR by inhibiting *XBP1* splicing, resulting in unresolved ER stress [20]. As shown in Fig. 2a, both *GRP78* expression and *XBP1* splicing activity are increased in human PDAC cells (Panc02.03) treated with 2.5 µM tunicamycin. *XBP1* splicing could be reduced to normal level when also treated with STF-083010 at 25 µM or above, suggesting that PDAC cells are responsive to chemical activation and inhibition of the ER stress-UPR pathway. STF-083010 treatment did not alter the tunicamycin-induced elevated expression of *GRP78*, suggesting its specificity to IRE1α arm of UPR (Fig. 2a).

As it is hypothesized that ER stress regulates autophagy and lysosomal activity via arms of UPR pathway, we assayed autophagy in the tunicamycin and STF-083010 treated human PDAC cells (Panc02.03) by TEM and lysotracker staining. As shown by TEM analysis, both tunicamycin and STF-083010 significantly increased the number of matured lysosomes in the PDAC cells (*p* < 0.01, Fig. 2b). ER dilation, a hallmark feature of ER stress, was also frequently noticed in these treated cells (Fig. 2b). As evident in Fig. 2c, tunicamycin or STF-083010 increased the acidophilic lysotracker staining, suggesting increased lysosomal activities.

We used 4-PBA, a chemical chaperone that alleviates ER stress by augmenting protein folding and thereby reduces GRP78 expression, to examine its effect on lysosomal activity. Interestingly, 4-PBA could suppress both tunicamycin and STF-083010 induced upregulation of autophagy (Fig. 2c). This indicates that 4-PBA may be alleviating the accumulation of unfolded proteins thereby reducing ER stress and autophagy. This suggests that ER stress activates autophagy in PDAC cells, and that ER stress-UPR can be targeted to control cancer cell viability.

### ER stress and autophagy modulating compounds enhanced the anti-proliferative effects of gemcitabine in PDAC cells

Autophagy is one of the downstream UPR effect by which cells degrade/recycle excess unfolded proteins and defective organelles as a pro-survival adaptive mechanism. We examined the effect of inhibiting autophagy by chloroquine on the growth and survival of PDAC cells. Chloroquine, a well-known autophagy inhibitor, functions by preventing endosomal acidification, which inhibits both fusion of the autophagosome and lysosome, and lysosomal enzyme mediated degradation during the late autophagy process [29]. Lysotracker staining was diminished in chloroquine treated cells consistent with autophagy inhibition (Fig. 2c).

We then proceeded to evaluate the effect of combining STF-083010 and chloroquine with gemcitabine in several PDAC cell lines. The IC50 concentration for each of these drugs were derived and an optimized drug combination regimen was developed below the respective IC50 range, prior to proceeding with combination therapy. For most of our assays, cells were inoculated and allowed to reach 50% confluence and then treated for 72 h to evaluate their yield and viability (Fig. 2d).

Administration of STF-083010 at 25 µM caused cytotoxicity as evidenced by significantly reduced cell viability compared to control (*p *< 0.05). Gemcitabine alone at 250 nM could also significantly reduce cell viability (*p *< 0.05). Co-administration of STF-083010 and chloroquine could further reduce the cell survival (*p *< 0.01), showing an additive effect (Fig. 2d). Similarly, additive effects were evident when STF-083010 and gemcitabine were co-administered (*p *< 0.01). Co-administering chloroquine, STF-083010 and gemcitabine together did not show significant reduction of cell viability, compared to dual treatment of gemcitabine and STF-083010 (*p* = 0.61). Although, STF-083010 can reduce PDAC cell viability in vitro, it is physiologically unstable and hence is not suitable for clinical development.

### Administration of sunitinib showed synergistic effects with gemcitabine in reducing viability of pancreatic cancer cell lines

Chemotherapeutics are often limited in their efficacy as tumors progressively acquire chemoresistance. ER stress-induced autophagy is believed to be an important mechanism through which tumors develop chemoresistance. Hence, we sought to compare the utility of clinically approved drugs that may affect the autophagy/lysosomal processes. Sunitinib is a multi-tyrosine kinase inhibitor that also targets VEGF receptors, PDGF receptors and KIT, thus disrupting angiogenesis [30]. Sunitinib has been approved for the treatment of metastatic renal carcinoma, gastrointestinal stromal tumor (GIST) and pancreatic neuroendocrine tumor [31, 32]. In addition, it was hypothesized that sunitinib may alter the autophosphorylation and activity of IRE1α, as well as lysosomal enzymatic activity [21, 22, 33, 34]. Hence, we chose to use sunitinib to assess its effects in altering the ER stress, autophagy and tumor response.

**Fig. 3:**
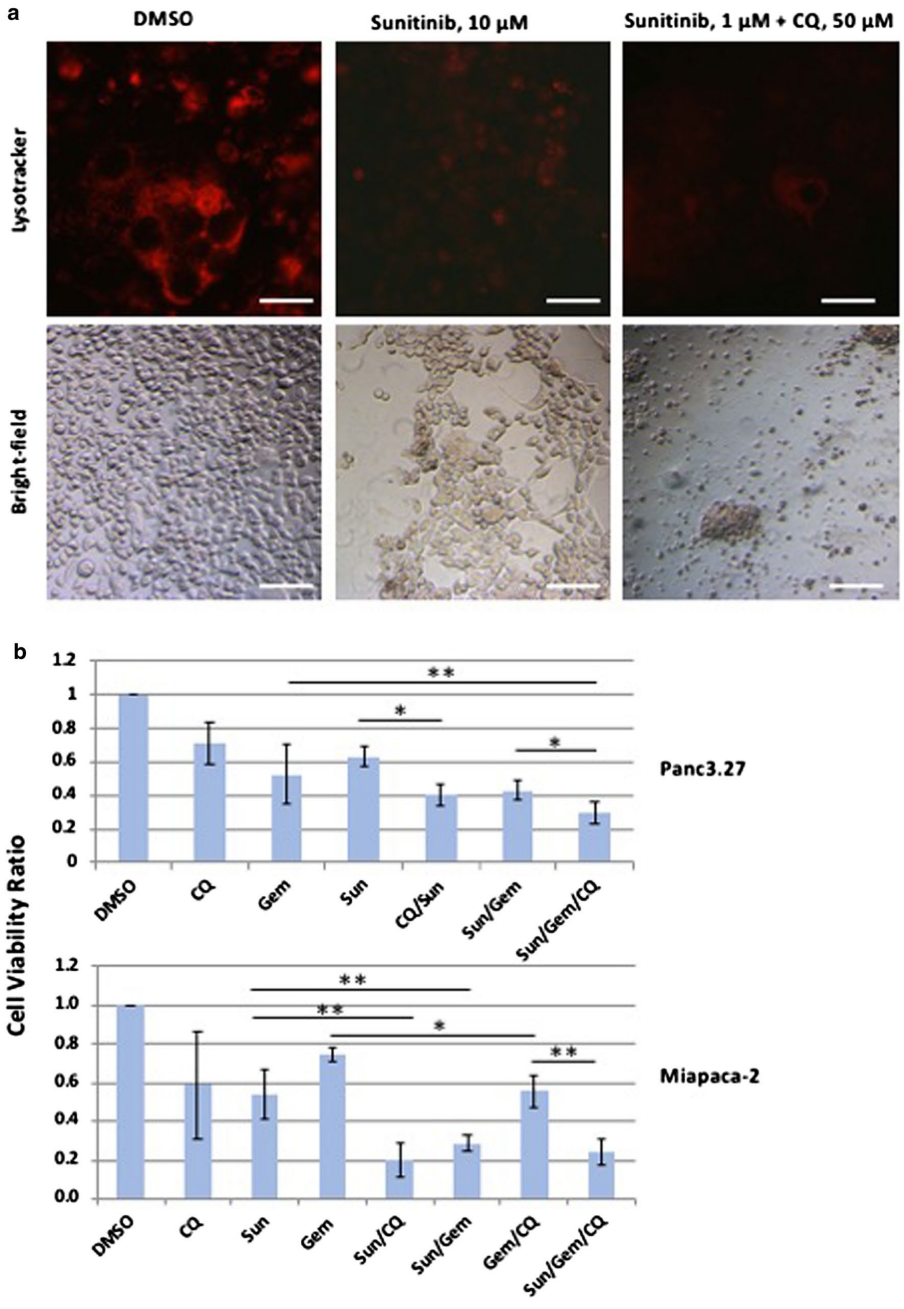
Additive effects of sunitinib with chloroquine and gemcitabine on growth and survival of human PDAC cells. **a** Lysotracker staining showing lysosomal activity in DMSO, sunitinib, and combination treatments (top panel), and the corresponding bright field image (bottom panel). Panc3.27 cells were treated with sunitinib (10 µM), chloroquine (25 µM) and gemcitabine (100 nM) for 72 h and imaged with fluorescent (top panel) and bright-field microscopy (bottom panel). Scale bar: 10 µm. **b** Bar-charts showing normalized cell viability of Panc3.27 and Miapaca-2 PDAC cells treated with sunitinib, chloroquine, gemcitabine and various combinations (n = 5). The cell viability was measured by Trypan Blue exclusion assay following 72 h of drug treatment. The combination treatments resulted in significantly decreased cell viability for Sun/CQ (*p* < 0.05), Sun/Gem (*p* < 0.01) and Sun/Gem/CQ (*p* < 0.01) treatments in Panc3.27 and Miapaca-2 cells. The triplet combination of Sun/Gem/CQ could reduce cell viability more than threefold in both cell lines. Cell viability ratio was normalized with the viability of DMSO treated cells. *p < 0.05, **p < 0.01, *CQ* chloroquine, *Gem* gemcitabine, *Sun* sunitinib

After a dose response study, an IC50 and optimal dosing regimen was derived for sunitinib in Panc3.27 and Miapaca-2 cells. As shown in Fig. 3a, sunitinib-treated cells showed reduced growth. Co-administration of sunitinib significantly enhanced the cytocidal effect of gemcitabine in both Panc3.27 and Miapaca-2 cell lines (*p* < 0.01, Fig. 3b). Sunitinib and chloroquine combination exhibited an additive effect without gemcitabine (*p* < 0.01, Fig. 3b). Interestingly, Miapaca-2 cells showed a greater sensitivity to chloroquine and sunitinib compared to Panc3.27 cells (Fig. 3b).

We analyzed *XBP1* splicing and lysosomal function in sunitinib and chloroquine treated cells. Sunitinib did not affect the *XBP1* splicing (Additional file 2: Figure S2A), indicating it does not directly alter the IRE1alpha-mediated splicing activity. However, lysotracker staining was significantly diminished in sunitinib or chloroquine treated cells, suggesting that both are affecting the lysosomal degradation process.

### Sunitinib increases intracellular multivesicular lysosomes, reduces lysosomal degradation and increases apoptosis of pancreatic cancer cell lines

**Fig. 4:**
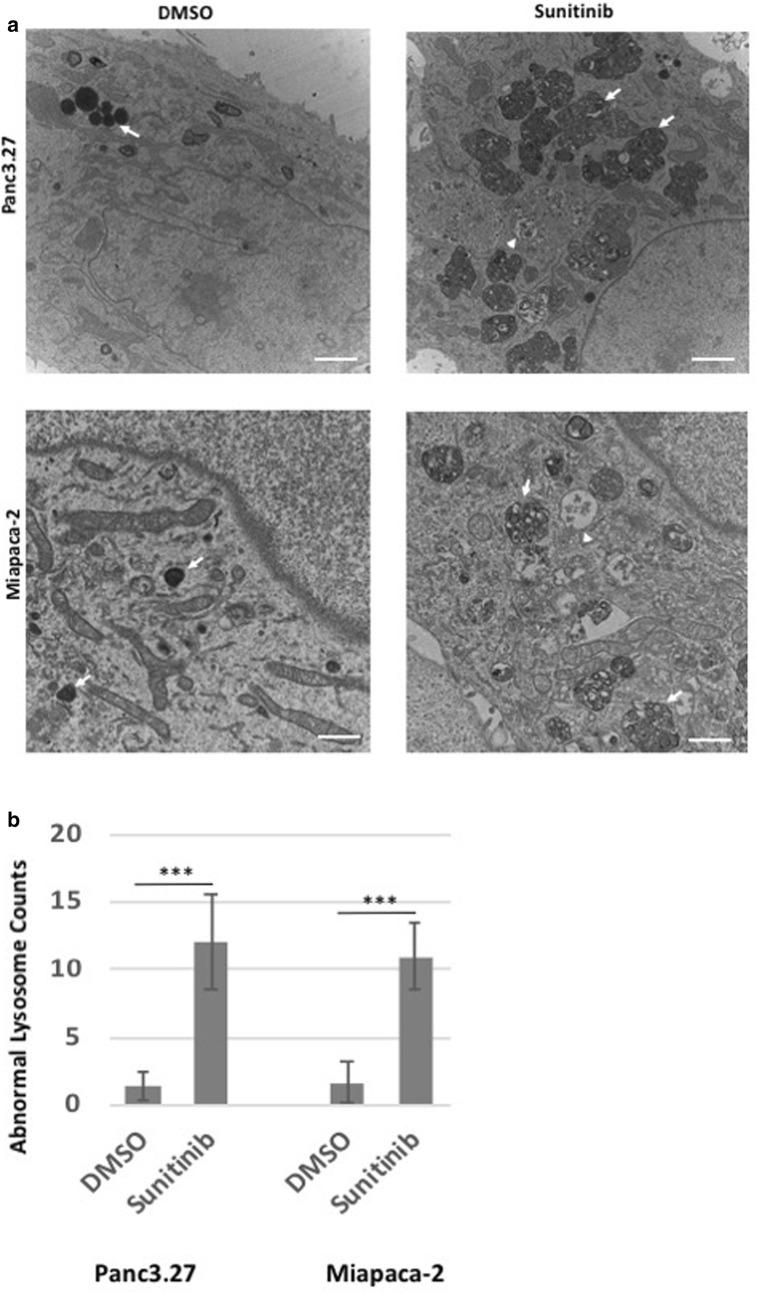
Ultrastructural pathology of sunitinib treated human PDAC cells. Panc3.27 and Miapaca-2 cells were treated with DMSO or sunitinib, 10 µM for 72 h and fixed in glutaraldehyde/PFA and processed for electron microscopy. TEM images of DMSO (left) and sunitinib-treated (right) Panc3.27 cells (top panel) and Miapaca-2 cells (bottom panel) showing organelle pathology of ER vesicles (arrowhead), and lysosomes (arrow). Sunitinib treated cells exhibit enlarged ER lumen with granular materials and have large multivesicular lysosomal bodies in the cytoplasm surrounding the nucleus. Scale bar, 500 nM. *CQ* chloroquine, *Gem* gemcitabine, *Sun* sunitinib

To examine further the effect of Sunitinib on cellular organelles, ER stress and autophagy, we performed ultrastructural analyses of PDAC cells by transmission electron micrography (TEM). In the control groups of both Panc3.27 and Miapaca-2 cell lines, normal organelles, normal ER structures, normal lysosomes and mitochondria were apparent (Fig. 4a). While matured lysosomes can be frequently seen in control cells, large incomplete multivesicular auto-lysosome bodies were abundantly seen in sunitinib-treated Panc3.27 and Miapaca-2 cells (*p* < 0.001, Fig. 4b). These multivesicular autolysosome bodies contained largely undigested materials, suggesting incomplete autophagy. Expanded ER lumen and macroautophagic vesicles were also observed in sunitinib-treated cells. These data suggest that sunitinib may inhibit late-stage autophagy and lysosomal degradation leading to apoptotic cell death.

**Fig. 5:**
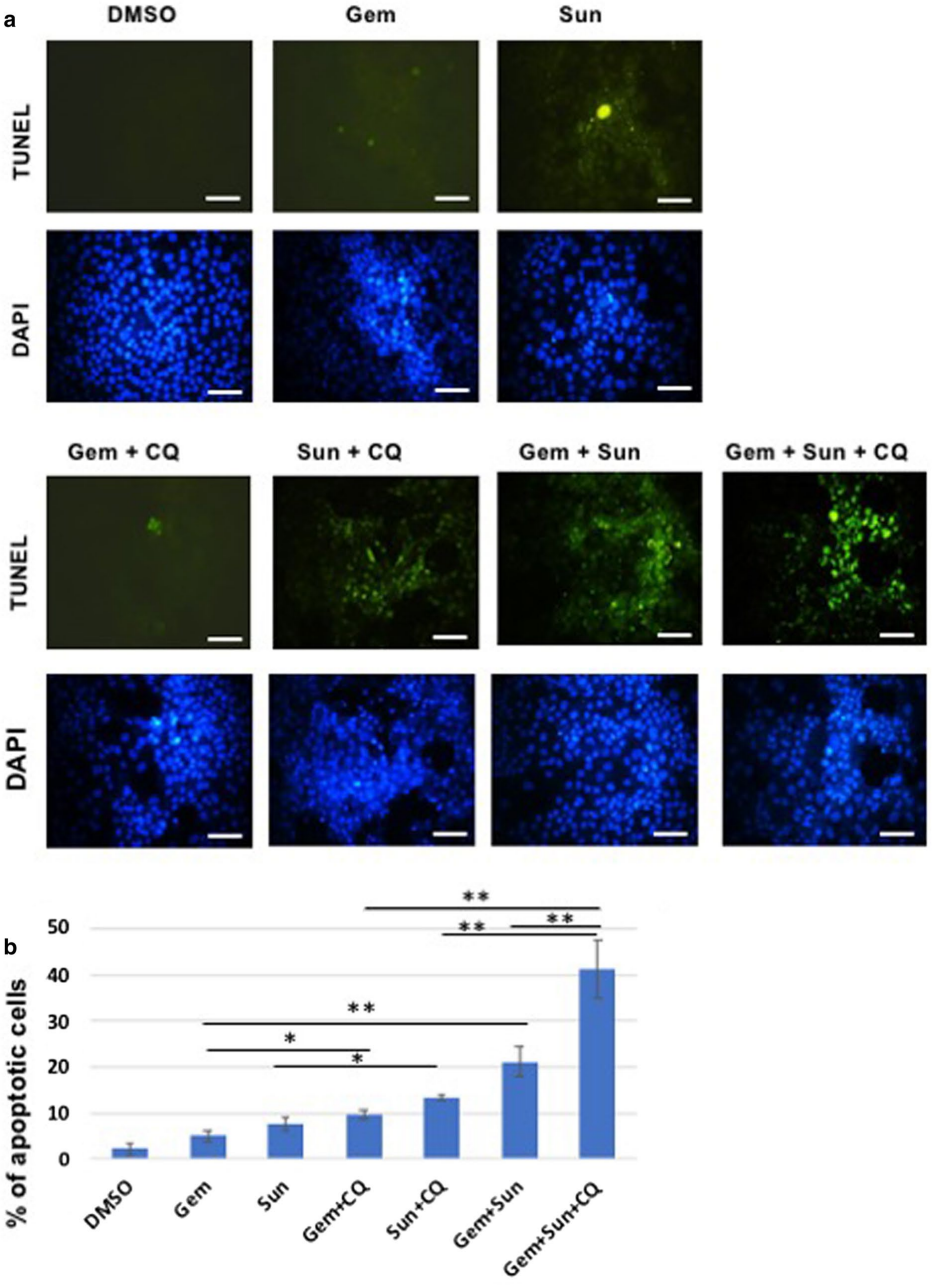
TUNEL staining showing synergy of sunitinib and gemcitabine in apoptosis of human PDAC cells. **a** Miapaca-2 were cultured in 4-well sterile borosilicate chambered cover glass and treated for 48 h with gemcitabine (250 nM), sunitinib (10 µM), and chloroquine (25 µM), or in combinations. Cells were fixed and processed for TUNEL and DAPI staining and were visualized using a fluorescent microscope. The combination treatment shows significantly increased TUNEL positive cells compared to the single treatment groups. **b** Bar-charts showing mean percentage of TUNEL positive cells in each treatment groups (n = 5). The combination treatments result in significantly increased TUNEL positive cells for Sun + CQ (*p* < 0.05), Gem + CQ (*p* < 0.05), Sun + Gem (*p* < 0.01) and Sun + Gem + CQ treatments (*p* < 0.01). *p < 0.05, **p < 0.01, Scale bar, 5 µM, *CQ* chloroquine, *Gem* gemcitabine, *Sun* sunitinib

TUNEL staining, which demonstrates DNA fragmentation seen in apoptosis revealed only moderately increased apoptosis in gemcitabine alone treatment compared to the DMSO treatment (*p* = 0.062, Fig. 5), whereas all other drug treatment groups showed significantly increased apoptosis (*p* < 0.05). Within the treatment groups, sunitinib treatment alone caused similar level of apoptosis as seen in the gemcitabine + chloroquine treatment groups. Significantly increased (*p* < 0.05) apoptosis was noticed in sunitinib + chloroquine, as well as in gemcitabine + sunitinib treated cells (*p* < 0.01, Fig. 5). The highest rate of apoptosis though was noticed in the tripslet combinations of gemcitabine + sunitinib + chloroquine (eightfold increase, *p* < 0.01).

### Co-administration of sunitinib with gemcitabine reduces in vivo tumor burden and increases survival in orthotopic murine PDAC models

To examine the efficacy of sunitinib co-administration with conventional chemotherapeutics, we analyzed its effects in relation to tumor progression and survival when administered in orthotopically transplanted Panc02 or KPCP1 murine tumor models. Panc02 cell lines carry an induced mutation in *K*-*ras* alone, while KPCP1 cell lines carry putative mutations in both *K*-*ras* and *P53*.

Panc02 or KPCP1 cells were directly injected into the pancreatic tail of female C57BL/6 mice via mini laparotomy incision and were allowed to grow in vivo. These orthotopic mice developed pancreatic cancer with 100% penetrance by 2–3 weeks post-transplantation. Mice with advanced tumors after 2-weeks post-transplantation were treated by daily oral administration of chloroquine, intra-peritoneal injection with sunitinib and/or gemcitabine + paclitaxel (Chemo) and control vehicle. For survival analysis, mice were followed until mortality by predetermined guidelines in accordance with the institutional animal protocol. For tissue corollary assays, mice were sacrificed at 4-week post-treatment and the pancreatic tissues were dissected to analyze histological features, proliferation, apoptosis and UPR marker expression.

**Fig. 6:**
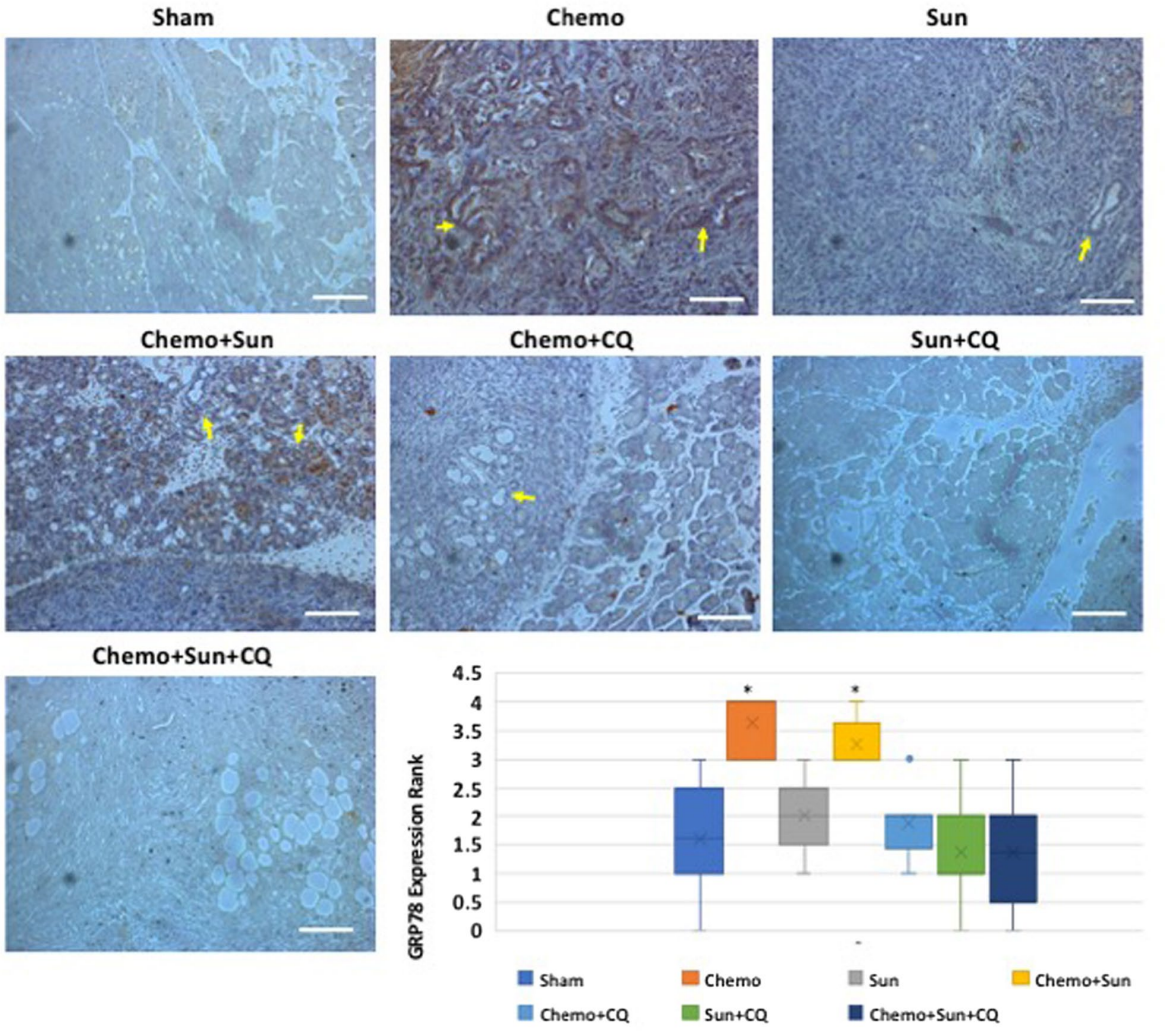
Anti-Grp78 immunohistochemistry showing differential expression of Grp78 in the pancreas of drug treated orthotopic murine model: Treatment with gemcitabine plus paclitaxel (Chemo) or Chemo + sunitinib increases the Grp78 levels in the ductal carcinoma (arrows) and surrounding cells (*p* < 0.05, Mann–Whitney *U*-test). This increased level of expression is reduced to basal level when Chemo was administered in combination with chloroquine, and sunitinib. While sunitinib alone also elevates Grp78 expression, a combination with Chloroquine reduces the elevated Grp78 to basal level. The intensity of Grp78 staining was ranked from 1 to 4 as weak, moderate, strong and very strong as shown in the bar-chart. *p < 0.05. Scale bar: 10 µm

**Fig. 7:**
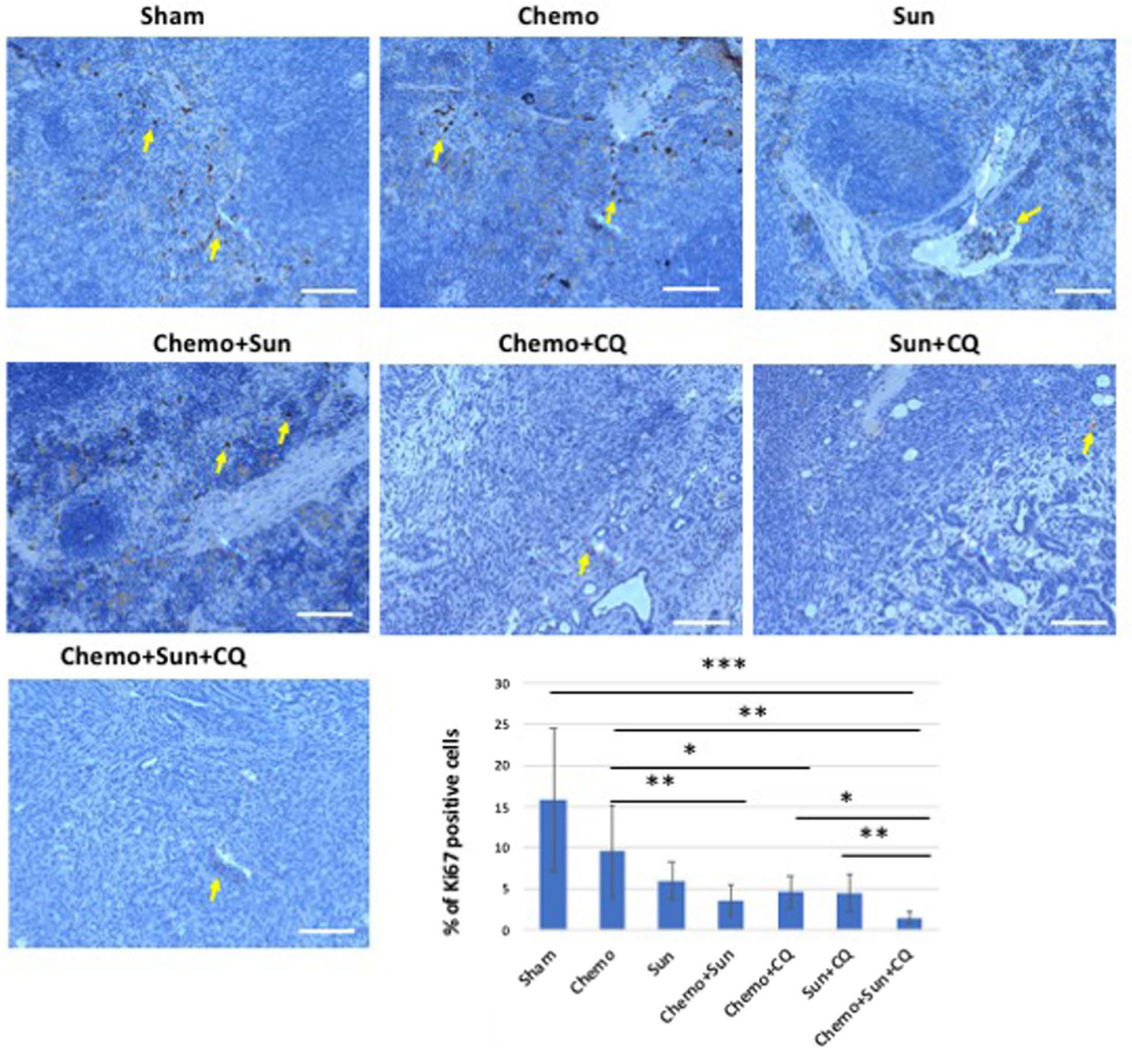
Anti-Ki67 immunohistochemistry showing differential reduction of cell proliferation in the pancreas of drug treated orthotopic murine model: The murine PDAC tissue from the control group shows significant proportion of Ki67 positive proliferating cells (arrow) in the pancreatic tissue. Although the Chemo alone does not reduce cell proliferation significantly (*p* = 0.16), the sunitinib treatment and all the combinatorial treatments significantly reduces the cell proliferation as shown in the bar-chart (*p* < 0.01). The triplet treatment with Chemo/Sun/CQ shows significantly reduced proliferation compared to the sham group (eightfold, *p* = 0.009), as well as further twofold lower from the doublet treatment groups (*p* < 0.01). *p < 0.05, **p < 0.01. Scale bar: 10 µm

The combinatorial drug treatments reduced ductal carcinoma formation. Large focal necrotic areas were evident in tissues from the Chemo + sunitinib + chloroquine treatment cohort (Additional file 2: Figure S2). The expression of the UPR sensor Grp78 was significantly upregulated in the PDAC tissues of mice treated with gemcitabine with or without sunitinib (*p* < 0.05, Fig. 6), whereas it was to the basal level of expression in the sunitinib + chloroquine and the Chemo + sunitinib + chloroquine cohorts (Fig. 6). To further examine tumorigenesis, we performed anti-Ki67 IHC on the pancreatic tissues of the treated and control mice and analyzed the cell proliferation. As expected, the proportion of Ki67 expressing cells were significantly reduced in all the combination treatment groups compared to the control (Fig. 7). There was also a statistically significant reduction in cell proliferation in sunitinib alone treatment (*p *= 0.044), although the gemcitabine alone treatment did not have significant effect (*p *= 0.16). There was no significant difference in cell proliferation between sunitinib + chloroquine and Chemo + sunitinib and the Chemo + chloroquine treatments (*p* = 0.113). However, the triplet combination of Chemo + sunitinib + chloroquine showed the highest reduction of cell proliferation compared to any other single or double treatment groups (*p* < 0.01).

**Fig. 8:**
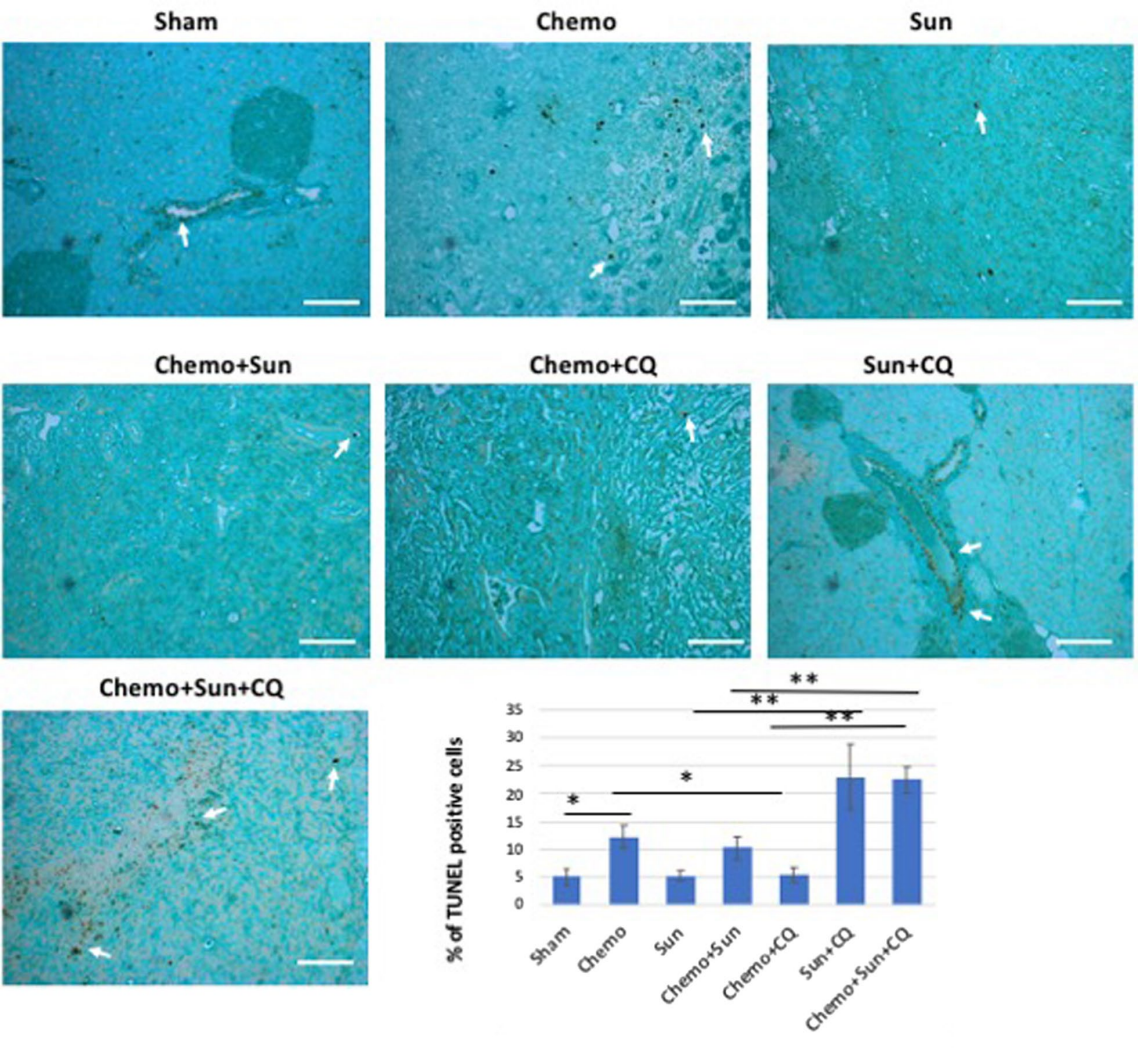
TUNEL staining showing differentially increased apoptosis in the pancreas of drug treated orthotopic murine model: The murine PDAC tissue from the control group shows the lowest proportion of TUNEL positive cells (arrow) in the pancreatic tissue. Both the sunitinib/chloroquine doublet treatment and the Chemo/sunitinib/chloroquine triplet treatment robustly increases the apoptotic cells in the ductal carcinoma region (six to sevenfold increase, *p* = 0.0017 and 0.0037, respectively). As shown in the bar-chart, the chemo alone, and Chemo/sunitinib doublet treatment also increase apoptosis significantly (two to threefold increase, *p* < 0.05), whereas sunitinib and the Chemo/chloroquine treatment do not alter apoptosis significantly (*p* = 0.79 and 0.53, respectively). *Sham* vehicle control, *Chemo* gemcitabine plus paclitaxel, *Sun* sunitinib, *CQ* chloroquine. *p < 0.05, **p < 0.01. Scale bar: 10 µm

We performed TUNEL assays on these tissue samples to quantify the apoptosis related cell death. Both the Chemo + sunitinib + chloroquine and sunitinib + chloroquine treatment groups had high proportion of TUNEL positive cells in the pancreatic tissue, especially in the ductal epithelium (*p* < 0.01, Fig. 8). Although, the Chemo alone and Chemo + sunitinib group also showed significant apoptosis (*p* < 0.05), there was no significant increase in apoptosis in the sunitinib, or Chemo + chloroquine groups in this assay (*p* = 0.18 and 0.22 respectively). We further validated the cellular apoptosis by examining the levels of active Casp3, a well-known early indicator of apoptosis. Anti-active Casp3 IHC showed increased Casp3 activity in Chemo + CQ, Sun + CQ and Chemo + Sun + CQ in significantly increasing order (*p *< 0.05, Additional file 3: Figure S3). The triplet combination of Chemo + Sun + CQ showed significantly more proportion of active Casp3 positive PDAC cells compared to the either of the doublet treatments Chemo + Sun (*p* < 0.01), Chemo + CQ (*p* < 0.01) or Sun + CQ (*p* < 0.05).

**Fig. 9:**
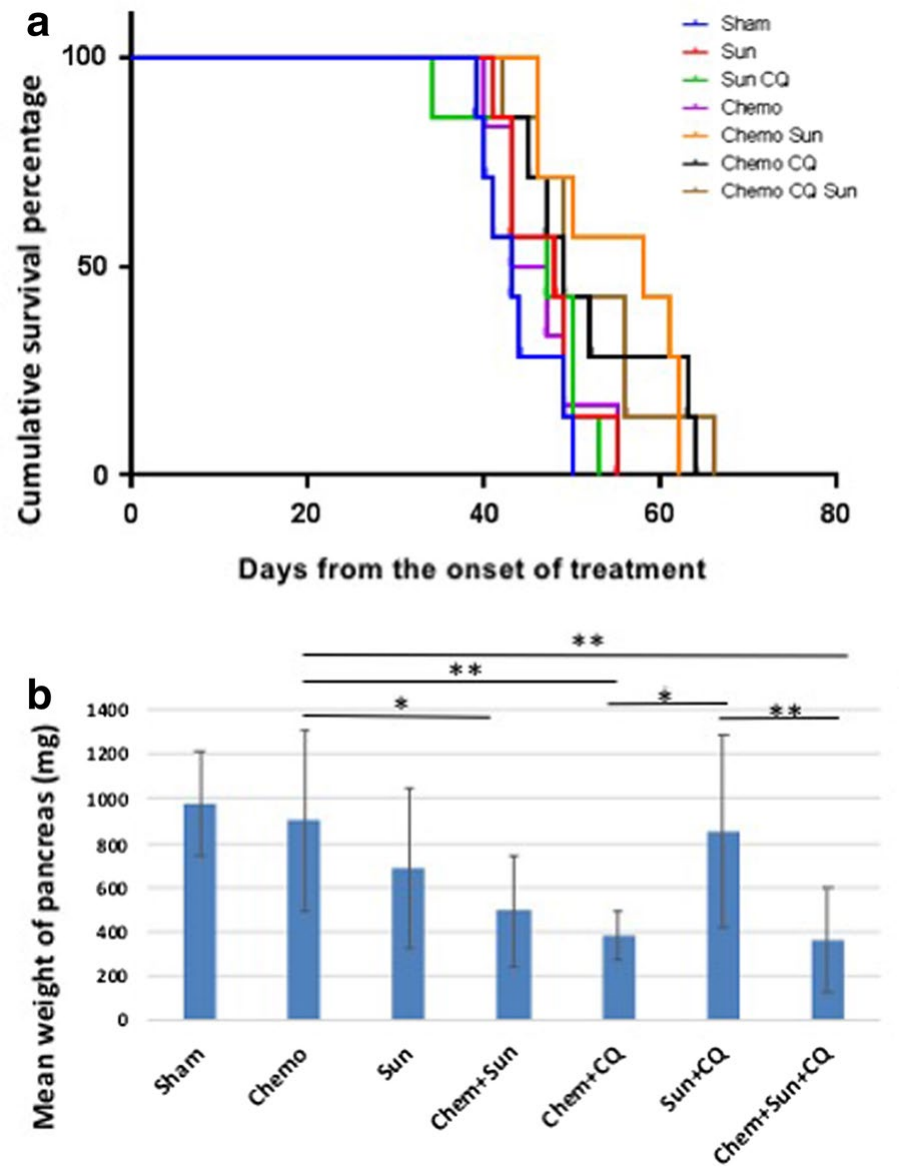
Survival analysis of Sunitinib, Chloroquine and Gemcitabine combinatorial treatment on orthotopic KPCP1 murine models in vivo. **a** Survival proportions of mouse PDAC models with KPCP1 cell implantation into the pancreatic tissue. Mice exhibiting clincial PDAC growth were treated with various combinations of sunitinib (Sun), chloroquine (CQ) and gemcitabine/paclitaxel (Chemo). The triplet combination shows highest survival rate (*p* < 0.001). The doublet combination treatments of Chemo + Sun, Chemo + CQ showed significant increase (*p* < 0.01) in survival compared to single treatment groups, while the Sun + CQ groups did not show significant increase in survival rate compared to either sham or single treatment groups. **b** Bar-charts showing mean pancreas weight after 4-weeks post-treatment in various treatment groups. The pancreas weight is significantly reduced in the Chemo + Sun (*p* < 0.05), Chemo + CQ (*p* < 0.01) and Chemo + Sun + CQ (*p* < 0.001) treatment groups, compared to Chemo or Sun alone treatment groups, while the monotherapy treatments or Sun + CQ treatment did not show any significant reduction in pancreatic mass. *Sham* vehicle control, *Chemo* gemcitabine plus paclitaxel, *Sun* sunitinib, *CQ* chloroquine

In the KPCP1 orthotopic cohort, treatment with Chemo alone, sunitinib alone, or sunitinib + chloroquine did not cause any significant increase in survival compared to the control group, as determined by Kaplan–Meier curve analyses (Fig. 9a). The survival of mice undergoing combination therapy of Chemo + sunitinib, Chemo + chloroquine and Chemo + sunitinib + chloroquine showed a significant increase of survival (*p* < 0.05, Fig. 9a). These treatment groups also showed significantly increased survival in the Panc02 orthotopic cohort (*p* < 0.01, Additional file 3: Figure S3). These three treatment groups were able to survive an average 2 weeks longer compared to the control groups in the KPCP1 cohort (63 ± 3 days vs 48 ± 4 days). The reduction of pancreatic tumor growth was also evident from a significant reduction of pancreatic weight after 4-weeks of drug treatments in the dual treatments of Chemo + sunitinib (*p* < 0.05), Chemo + chloroquine (*p* < 0.01) and the triplet combination of Chemo + sunitinib + chloroquine (*p* < 0.01) as shown in Fig. 9b. Either of monotherapy with Chemo or sunitinib and the dual therapy of sunitinib + chloroquine did not result in any significant reduction of pancreatic mass (Fig. 9b).

The mean survival rate was much higher in the Panc02 cohort, with the Chemo + sunitinib + chloroquine group showing an increase in maximum survival of more than 68 days (90% increase) as compared to the control group (115 ± 26 days vs 47 ± 11 days, Additional file 4: Figure S4). The Chemo + chloroquine (99 ± 22 days), and Chemo + sunitinib group (87 ± 19 days) also survived significantly longer compared to the control or single treatment groups in the Panc02 model (*p* < 0.05, Additional file 4: Figure S4). While gemcitabine treatment alone did not increase survival in the KPCP1 cohort, as compared to the control group (52 ± 2 vs 48 ± 4 days), it was significantly effective in the Panc02 cohort (80 ± 26 days vs 47 ± 11 days, Additional file 4: Figure S4). The sunitinib + chloroquine combination did not significantly increase survival compared to either sham groups or sunitinib treatment group in either KPCP1 or Panc02 orthotopic model (*p* = 0.32 and 0.43 respectively). Thus, the results from the survival analysis suggest that Chemo + sunitinib + chloroquine treatment significantly increases survival of the mice developing PDAC derived from either KPCP1 or Panc02 cells. Furthermore, dual treatments of both Chemo + chloroquine and Chemo + sunitinib also offers therapeutic opportunities in increasing survival rate.

Our data shows that sunitinib alone was sufficient to suppress cell growth, but does not increase cell death, and the sunitinib therapy alone was not sufficient to increase overall survival. However, sunitinib presents an effective therapeutic intervention if co-administered with gemcitabine and chloroquine in the murine PDAC models. Taken together, these results suggest that targeting multiple pathways of ER stress and autophagy is well tolerated by animals and these drug combinations increase the response of PDAC to chemotherapy and increases animal survival.

## Discussion

The incidence of pancreatic adenocarcinoma is rising in the US and is predicted to be the second leading cause of cancer related deaths in the US by 2020. Surgical intervention offers the only opportunity for a cure, but the prognosis of patients after surgery is still very poor [1]. Although some of the risk factors and genetic mutations associated with PDAC are known [35], these genes and molecular pathways are not easily targetable by available drugs. Therefore, understanding alternative molecular mechanisms that contribute to growth and survival of pancreatic adenocarcinoma would facilitate targeting of these pathways with potential for positive effects on treatment and prognosis. One of these molecular pathways is the ER stress pathway and lysosomal degradation believed to play a role in chemoresistance and tumor growth. We demonstrated that combining two agents targeting various aspects of this pathway, sunitinib and chloroquine to gemcitabine treatment, increases the efficacy over gemcitabine alone in reducing tumor growth through apoptosis and reduced proliferation. These findings suggest that modulators of ER stress, autophagy and lysosomal degradation, may be of utility in improving survival of patients with pancreatic cancer.

In our study, we determined that the proximal ER stress sensor GRP78 is actively expressed in the human PDAC tissues from resected specimens (Fig. 1). GRP78 expression is believed to be associated with cancer development and progression [36–39], and may serve as a prognostic marker correlated with disease status [40]. However, the correlation between GRP78 expression and the clinical pathological characteristics or prognosis of PDAC have not been well understood. Our IHC data reveal that GRP78 is expressed at significantly higher levels in the PDAC and stromal cells in comparison to the non-cancerous tissue within the diseased pancreas (Fig. 1a, c). This finding suggests that pancreatic cancer tissue is likely under ER stress and the UPR is triggered to restore ER homeostasis. We also observed mild upregulation of GRP78 in the histologically normal appearing tissues adjacent to the tumor compared to the pancreatic tissue biopsies from healthy individuals (Fig. 1c). A recent study has shown increased GRP78 expression in human PDAC tissue samples, in which high expression within normal pancreatic acinar cells around the PDAC tissue was also evident [19]. It is likely that elevated ER stress in the adjacent healthy tissue might be attributable to the tumorigenic and inflammatory stress from the surrounding PDAC tissue [41]. Additionally, we noticed similarly elevated GRP78 expression in the acinar cells and surrounding inflammatory cells in biopsies from pancreatitis patients (Fig. 1c), indicating a possible role of GRP78 in pancreatitis and in areas of pancreatic inflammation in general. GRP78 is highly expressed in many inflammatory diseases such as ulcerative colitis, and Crohn’s disease [42]. Consistent with these observation, our earlier studies revealed that GRP78 is robustly elevated in animal models of IBD and steatohepatitis exhibiting ultrastructural pathology of ER stress [43, 44].

There are contradictory findings on the prognostic significance of GRP78 in human cancers. For instance, high GRP78 expression was associated with decreased overall 5-year survival in gastric cancer [39] and hepatocellular carcinoma [45], whereas the opposite conclusion was found in colorectal cancer [46]. Therefore, inhibitors of GRP78 and other ER stress-UPR components, such as IRE1α, and downstream autophagy need to be studied within specific tumor types. Our data suggest that autophagy and UPR inhibitors could lead to an improved clinical response in PDAC.

In the murine orthotopic pancreatic model using KPCP1 cells, Grp78 is elevated upon gemcitabine treatment, and could be reduced to basal level when gemcitabine is co-administered with sunitinib and chloroquine (Fig. 6). This suggests that the UPR is often robustly elevated in PDAC tissues in response to chemotherapy-induced cellular stress and tumor cells may utilize the UPR as a survival mechanism. We performed in vitro experiments to analyze the role of ER stress modulators to alter the growth and survival of PDAC cells. Cell viability assays showed that Panc02.03, Panc3.27 and Miapaca-2 cells were sensitive to tunicamycin and STF-083010 (Fig. 2d). Both tunicamycin and STF-083010 treatment resulted in excessive level of lysosomes in these cell lines as seen by TEM and lysotracker staining (Fig. 2b, c). Although stress generally triggers a pro-survival response, persistent unresolved ER stress can switch the cytoprotective functions of UPR and autophagy into cell death [47, 48]. Chronic or acute ER stress can also lead to cell death due to the inability of cell to cope beyond a threshold of cellular stress and overwhelming autophagy [49]. In our previous studies in a zebrafish model of ER stress-mediated gastrointestinal inflammation, administration of 4-PBA, a chemical chaperone alleviated ER stress, reduced autophagy and grp78 expression and ameliorated inflammatory pathologies [43, 44]. In this study, 4-PBA reduced autophagy caused by tunicamycin in PDAC cells (Fig. 2c), suggesting the direct association of ER stress and autophagy in these cancer cells. Importantly, STF-083010 increased apoptosis, suggesting sensitivity of PDAC cells to modulation of IRE1α arm of UPR. In further experiments, synergism was found by combining STF-083010 with FDA approved agents such as gemcitabine, oxaliplatin and bortezomib (Fig. 2d and data not shown). Thus, selective inhibition of ER stress by IRE1α inhibitors could curb cancer cell growth and may increase the efficacy of several anti-tumor chemotherapeutics.

An important mechanism in restoring ER homeostasis is the removal of misfolded proteins, which can then be degraded by the ubiquitin proteasome system in the cytosol after translocation from the ER through the ERAD process [50]. However, an alternative pathway for degradation of ER proteins is via autophagy, which involves the sequestering of material that needs to be degraded through autophagosomes, followed by fusion with a lysosome and degradation by lysosomal enzymes. In our studies, inhibiting autophagy by chloroquine improves the efficacy of gemcitabine and sunitinib (Figs. 4, 5, 6, 7, 8, 9).

In these studies, the anti-tumor efficacy of STF-083010 suggested that in addition to autophagy, the UPR pathway is a promising target to treat PDAC. Sunitinib, among its many functions, is believed to increase the lysosomal pH and inhibition of the lysosomal protease activity [34]. In addition, it has been hypothesized that sunitinib may also alter the activity of IRE1α and that it is a lysosomotropic agent predominantly sequestered in lysosome compartments, presumably inhibiting lysosomal enzymatic activity [21]. Since the lysosomal degradation pathway is controlled by the constitutive UPR, we believe directly altering this sequela of ER stress using sunitinib and chloroquine may achieve similar outcomes as UPR inhibitors. In both our in vitro and in vivo studies, sunitinib increased the efficacy of gemcitabine and chloroquine (Figs. 3, 5, 6, 7, 8, 9, and Additional file 4: Figure S4). Ultrastructural analysis of sunitinib treated PDAC cells reveal features of ER expansion and large clusters of multivesicular lysosomal bodies with undigested materials (Fig. 4), indicating that in addition to its other metabolic effects, sunitinib also inhibits late stage autophagy and leads to defective auto-lysosomal degradation. The drastic reduction of lysotracker staining in sunitinib treated cells and the accumulation of large lysosomal bodies further indicates that sunitinib affects lysosomal degradation and contributing to cytotoxicity (Figs. 3, 4, 5). In fact, in our murine model, PDAC tissue exhibited significantly increased apoptosis and reduction in ductal cell growth upon treatment with sunitinib in combination with gemcitabine or chloroquine (Figs. 7 and 8).

In both the orthotopic murine models with in vivo intra-pancreatic transplantation of murine Panc02 and KPCP1 cells, sunitinib showed strong synergy with gemcitabine and chloroquine by significantly increasing tumor response and survival rate (Figs. 7, 8, 9 and Additional file 4: Figure S4). In our survival analysis, the triplet combination of Chemotherapy + sunitinib + chloroquine showed the highest survival rate. Survival was significantly higher than seen in all the monotherapy groups. as well as the combinations of chemotherapy + sunitinib and sunitinib + chloroquine groups (*p* < 0.05). In addition, the survival of those treated with the triplet combination was higher than the chemotherapy + chloroquine treatment groups with a *p*-value close to 0.05 (*p* = 0.054, Panc02 and *p* = 0.068, KPCP1 mouse models). We also note that the combination of gemcitabine + chloroquine showed a significantly increased survival and tumor response, compared to the monotherapy groups, as well to treatment with sunitinib + chloroquine (*p* < 0.05).

Similar to our findings in these preclinical studies, inhibition of autophagy with hydroxychloroquine alone does not show efficacy as a single agent in the clinical treatment of PDAC [51]. As PDAC tumors undergo increased ER stress upon chemotherapy treatment, we hypothesized the addition of autophagy inhibition to chemotherapy would improve PDAC response to therapy [52]. In previously reported clinical studies from our group, the addition of hydroxychloroquine to neoadjuvant chemotherapy improved clinical response parameters compared with chemotherapy alone [52]. From these current experiments, we further surmise that the concomitant inhibition of the lysosomal-UPR pathway by the addition of sunitinib to chloroquine, would lead to an improved clinical response. Plans to test this clinical hypothesis are underway.

## Conclusion

The development of combinatorial targeted cancer therapies is often motivated by the exploitation of tumor specific characteristics to increase treatment efficacy. PDAC tumors inherently have high levels of ER stress, which is further promoted by chemotherapy. These studies demonstrate that the ER stress-UPR-lysosomal pathway can be targeted to potentially improve the treatment of patients suffering from pancreatic adenocarcinoma. These data support clinical investigations of the combination of chemotherapy with agents such as hydroxychloroquine and sunitinib that target the ER stress, UPR and lysosomal pathways.

## Additional files

**Additional file 1: Figure S1.** Diagrammatic representation of ER stress and autophagy pathway and the drug targets that are used in this study to intervene the ER-autophagy pathway.

**Additional file 2: Figure S2.** (A) RT-PCR showing *XBP1-u* and *XBP1-s* expression levels in DMSO, tunicamycin, sunitinib and sunitinib+chloroquine treated Panc3.27 cells. Sunitinib or sunitinib+chloroquine treatment does not alter the expression of *XBP1-s*. (B) Histological analysis of mice treated with chemotherapeutics, sunitinib and chloroquine. The dual combination of Chemo+chloroquine, Chemo+sunitinib and the triple treatment of Chemo+sunitinib+gemcitabine shows noticeable reduction of ductal carcinoma (arrow). Scale bar: 10 µm. *Sham* vehicle control, *Chemo* gemcitabine plus paclitaxel, *Sun* sunitinib, *CQ* chloroquine.

**Additional file 3: Figure S3.** IHC staining with anti-active Casp3 showing differentially increased apoptosis in the pancreas of drug treated orthotopic murine model: The murine PDAC tissue from the control group shows the lowest proportion of active Casp3 positive cells (arrow) in the pancreatic tissue. Both the Sun+CQ, and Chemo+CQ doublet treatment and the Chemo+Sun+CQ triplet treatments robustly increases the apoptotic cells in the ductal carcinoma region (*p* = 0.037, 0.004, 0.0006, respectively). As shown in the bar-chart, neither sunitinib alone, nor chemo alone could alter apoptosis significantly (*p *= 0.089, 0.12 and 0.071, respectively). However, there was a statistically significant increase of active Casp3 positive cells in the triplet treatment group when compared to all of the double treatment groups of Chemo+Sun (*p* = 0.0007), Chemo+CQ (*p* = 0.008) and Sun+CQ (*p *= 0.019). *Sham* vehicle control, *Chemo* gemcitabine plus paclitaxel, *Sun* sunitinib, *CQ* Chloroquine. *: *p* < 0.05, **: *p *< 0.01. Scale bar: 10 µm.

**Additional file 4: Figure S4.** Survival analysis of Sunitinib, Chloroquine and Gemcitabine combinatorial treatment on orthotopic Panc02 murine models in vivo. **(A)** Cumulative survival **(B)** Mean overall survival. Mice exhibiting clincial PDAC growth were treated with various combinations of sunitinib (Sun), chloroquine (CQ) and gemcitabine/paclitaxel (Chemo). The triplet combination shows highest survival rate (*p* < 0.001). The Panc02 orthotopic model showed significantly increased mean survival for either of the single treatments of Chemo, Sun or CQ, compared to the sham groups (*p* < 0.05). Overall, the Panc02 model showed greater sensitivity to the combination drugs and longer survival compared to Kpcp1 models with the triplet combination resulting in longer than 4 months survival. *Sham* vehicle control, *Chemo* gemcitabine plus paclitaxel, *Sun* sunitinib, *CQ* Chloroquine. *: *p* < 0.05, **: *p *< 0.01.

## Abbreviations

PDAC: pancreatic ductal adenocarcinoma
ER: endoplasmic reticulum
UPR: unfolded protein response
Gem: gemcitabine
Sun: sunitinib
CQ: chloroquine
