# Supplementary figures and images for: Inhibition of endoplasmic-reticulum-stress-mediated autophagy enhances the effectiveness of chemotherapeutics on pancreatic cancer
Source: J Transl Med. 2018 Jul 9;16:190. doi: 10.1186/s12967-018-1562-z (PMC6038181; doi:10.1186/s12967-018-1562-z)

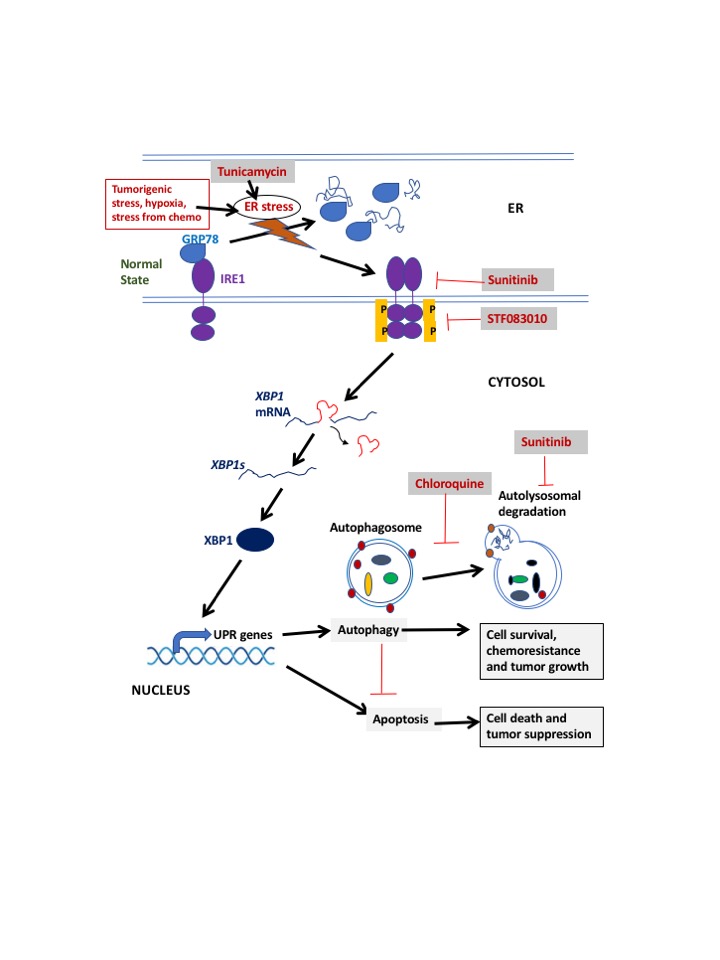

Supplement: Supplementary file 1 — Additional file 1: Figure S1. Diagrammatic representation of ER stress and autophagy pathway and the drug targets that are used in this study to intervene the ER-autophagy pathway. [file 12967_2018_1562_MOESM1_ESM.jpg]

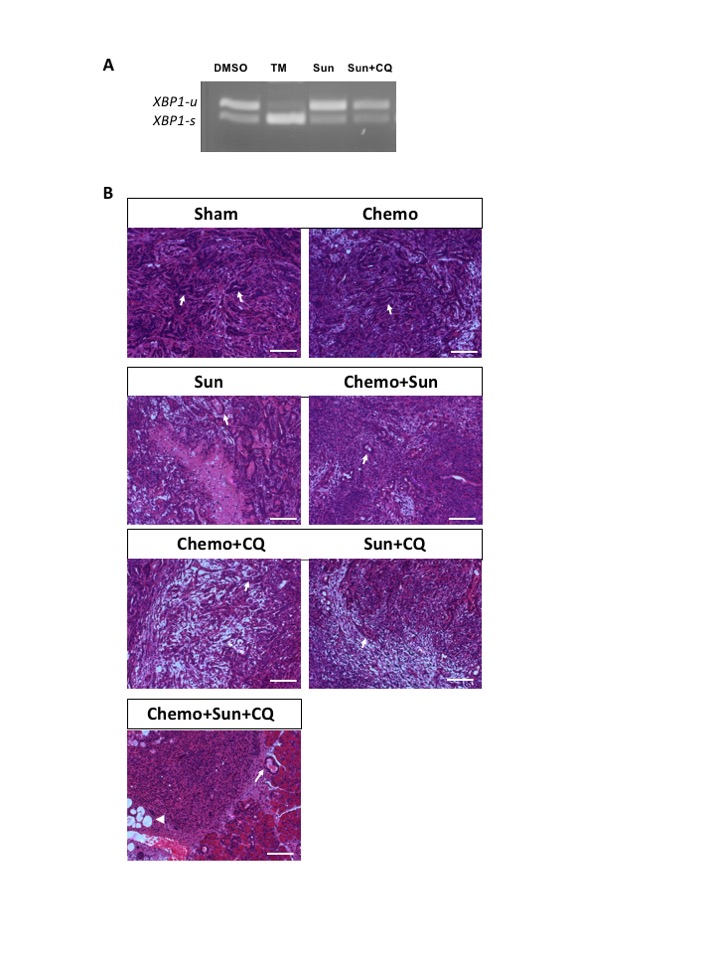

Supplement: Supplementary file 2 — Additional file 2: Figure S2. (A) RT-PCR showing XBP1-u and XBP1-s expression levels in DMSO, tunicamycin, sunitinib and sunitinib+chloroquine treated Panc3.27 cells. Sunitinib or sunitinib+chloroquine treatment does not alter the expression of XBP1-s. (B) Histological analysis of mice treated with chemotherapeutics, sunitinib and chloroquine. The dual combination of Chemo+chloroquine, Chemo+sunitinib and the triple treatment of Chemo+sunitinib+gemcitabine shows noticeable reduction of ductal carcinoma (arrow). Scale bar: 10 µm. Sham vehicle control, Chemo gemcitabine plus paclitaxel, Sun sunitinib, CQ chloroquine. [file 12967_2018_1562_MOESM2_ESM.jpg]

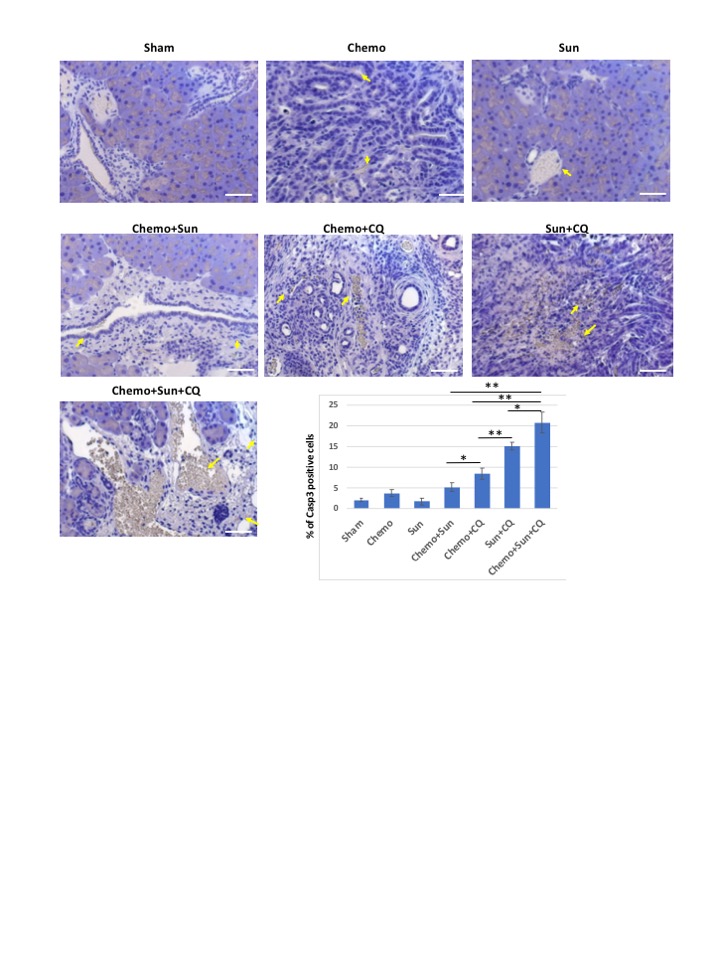

Supplement: Supplementary file 3 — Additional file 3: Figure S3. IHC staining with anti-active Casp3 showing differentially increased apoptosis in the pancreas of drug treated orthotopic murine model: The murine PDAC tissue from the control group shows the lowest proportion of active Casp3 positive cells (arrow) in the pancreatic tissue. Both the Sun+CQ, and Chemo+CQ doublet treatment and the Chemo+Sun+CQ triplet treatments robustly increases the apoptotic cells in the ductal carcinoma region (p = 0.037, 0.004, 0.0006, respectively). As shown in the bar-chart, neither sunitinib alone, nor chemo alone could alter apoptosis significantly (p = 0.089, 0.12 and 0.071, respectively). However, there was a statistically significant increase of active Casp3 positive cells in the triplet treatment group when compared to all of the double treatment groups of Chemo+Sun (p = 0.0007), Chemo+CQ (p = 0.008) and Sun+CQ (p = 0.019). Sham vehicle control, Chemo gemcitabine plus paclitaxel, Sun sunitinib, CQ Chloroquine. *: p < 0.05, **: p < 0.01. Scale bar: 10 µm. [file 12967_2018_1562_MOESM3_ESM.jpg]

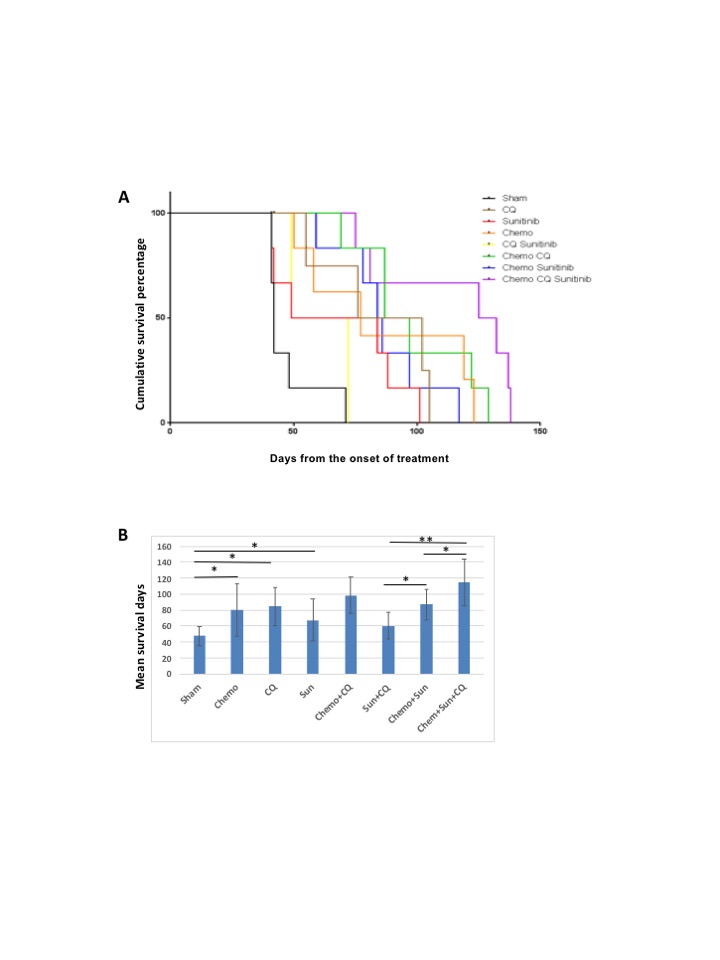

Supplement: Supplementary file 4 — Additional file 4: Figure S4. Survival analysis of Sunitinib, Chloroquine and Gemcitabine combinatorial treatment on orthotopic Panc02 murine models in vivo. (A) Cumulative survival (B) Mean overall survival. Mice exhibiting clincial PDAC growth were treated with various combinations of sunitinib (Sun), chloroquine (CQ) and gemcitabine/paclitaxel (Chemo). The triplet combination shows highest survival rate (p < 0.001). The Panc02 orthotopic model showed significantly increased mean survival for either of the single treatments of Chemo, Sun or CQ, compared to the sham groups (p < 0.05). Overall, the Panc02 model showed greater sensitivity to the combination drugs and longer survival compared to Kpcp1 models with the triplet combination resulting in longer than 4 months survival. Sham vehicle control, Chemo gemcitabine plus paclitaxel, Sun sunitinib, CQ Chloroquine. *: p < 0.05, **: p < 0.01. [file 12967_2018_1562_MOESM4_ESM.jpg]
